# Assessing adherence factors in patients under topical treatment: development of the Topical Therapy Adherence Questionnaire (TTAQ)

**DOI:** 10.1007/s00403-014-1446-x

**Published:** 2014-02-08

**Authors:** Ina Zschocke, Ulrich Mrowietz, Annett Lotzin, Eleni Karakasili, Kristian Reich

**Affiliations:** 1 SCIderm GmbH, Drehbahn 1-3, 20354 Hamburg, Germany; 2 Department of Dermatology, Psoriasis-Center, University Medical Center Schleswig-Holstein, Schittenhelmstraße. 7, 24105 Kiel, Germany; 3 Dermatologikum Hamburg, Stephansplatz 5, 20354 Hamburg, Germany

**Keywords:** Topical therapy, Compliance, Adherence, Psoriasis, Questionnaire, TTAQ

## Abstract

Medication adherence rates strongly depend on favorable disease outcomes. It is known that medication adherence rates are lower for topical treatment than for systemic treatment. However, to date no validated instrument for the assessment of adherence factors in topical treatment is available. The aim of this study was to develop a new questionnaire to assess adherence risk factors in topical treatment. The development of the Topical Therapy Adherence Questionnaire (TTAQ) and Patient Preference Questionnaire (PPQ) was based on a systematic literature review, and qualitative patient focus interviews and expert focus groups’ input. The psychometric properties and comprehensibility of the TTAQ and PPQ were assessed in a feasibility study with 59 psoriasis patients. Our first preliminary results indicate that the TTAQ and PPQ are psychometrically sound and reliable measures for the assessment of factors influencing topical treatment adherence. The questionnaires are currently being further developed and various parameters (e.g., time point of assessment) are currently being tested in an exploratory pilot study with ca. 2,000 psoriasis patients receiving topical treatment in a European clinical trial. The use of the final versions of TTAQ and PPQ in clinical practice may facilitate the early identification of specific non-adherence factors in patients under topical treatment, which could enable designing and applying adherence-enhancing interventions according to the patient’s individual needs.

**Electronic supplementary material:**

The online version of this article (doi:10.1007/s00403-014-1446-x) contains supplementary material, which is available to authorized users.

## Introduction

The WHO defines adherence to treatment as “the extent to which a person’s behavior—taking medication, following a diet, and/or executing lifestyle changes—corresponds with agreed recommendations from a health care provider.” [43] For the past three decades the importance of adherence has been recognized and proposed as a key factor in achieving the therapeutic goals of medical care [16]. The phrase of C. Everett Koop “Drugs don’t work in patients who don’t take them” [13] highlights the interaction and close relationship between patient adherence and desired treatment outcome [14]. A meta-analysis of 63 studies revealed that adherence reduces the risk for none or poor treatment outcomes by 26 % and increases threefold the probability of a good treatment outcome [16]. Importantly, poor medication adherence compromises safety and effectiveness of treatment, leads to increased morbidity and death and to increased direct and indirect costs for the health-care system [13, 15, 30, 35, 38].

According to its 2003 report the WHO considers adherence rates in developed countries to average only to about 50 % [43]. Within the last three decades a number of studies have reported that as many as 40 % of the patients fail to adhere to treatment recommendations while the percentage of non-adherent patients increases to 70 % when treatment regimens are too complicated and/or require lifestyle changes and modification of existing habits (reviewed in [34]). Patients with acute conditions are reported to be more adherent than the ones with chronic conditions whose persistence is very low and is markedly reduced after the first 6 months of treatment [38].

A number of factors have been identified as influencing long-term medication adherence such as the complexity, duration and cost of the treatment, condition characteristics (chronicity, severity, complicating factors), immediacy of beneficial or adverse effects, communication and information flow between the patient and the physician, socio-economic variables (health literacy, substance use disorders), concomitant multiple medication, patients’ beliefs on the necessity of the treatment as well as patients’ previous treatment experiences and expectancies from and satisfaction with the current treatment [10, 13, 28, 29].

Adherence to topical treatment has been found challenging since application of topical medications is often considered and reported by the patients as being more difficult than simply taking a pill [20]. Therefore, when assessing topical treatment adherence one has to consider additional specific aspects such as the cosmetic and galenic properties (very greasy, desiccating or sticky vehicles) and the smell of the preparation, the time required for its application as well as the convenience of application [4]. It is therefore not surprising that patients commonly consider topical treatment as unpleasant and time consuming and are commonly reporting their non-adherence to the recommended treatment [32]. However, non-adherence seems to be an even greater problem than patients would like to admit since electronic monitoring of patients’ controlled adherence behavior reveals that patients tend to overstate their use of medication and hence their adherence in treatment logs [20, 32]. In general, topical treatment adherence for dermatological conditions is poor, with primary adherence—prescription redemption—being only 65 % (for psoriasis patients primary adherence is 50 %) and secondary adherence—following prescribed treatment—ranging from 50 to 60 % [4, 20, 45].

In spite of these increased reported topical treatment non-adherence rates, only a few studies have attempted to investigate and identify why patients with dermatologic conditions fail to follow topical medication recommendations [12].

To date there is no reliable self-reporting tool for assessing adherence-influencing factors in patients under topical treatment. The primary goal of this study was to develop a novel tool termed Topical Therapy Adherence Questionnaire (TTAQ) which could allow physicians to identify potential factors for non-adherent behaviors at an early stage thus enabling the application of adherence-enhancing interventions according to each patient’s individual needs. Additionally, this study aimed at assessing the psychometric properties, comprehensibility and feasibility of the preliminary version of the TTAQ in a feasibility check with psoriatic patients under topical treatment.

## Methods

**Fig. 1:**
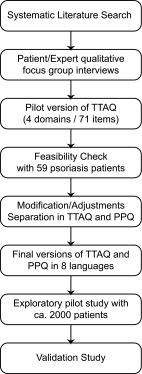
Development steps of the TTAQ and PPQ questionnaires

A schematic representation of the methodology for the development, initial feasibility check and future evaluation of the TTAQ and the Patient Preference Adherence Questionnaire (PPQ) is illustrated in Fig. 1.

### Development of the item pool

In order to ensure the content validity for the newly developed tool, an extensive literature search in combination with qualitative interviews with patient and expert focus groups was performed.

### Literature search

A systematic literature search of the Medline database was conducted in January and February of 2011. The aim was to identify existing self-reporting tools, i.e., tools used as patient reported outcomes for the assessment of aspects known/expected to influence adherence. Tools which assessed patient treatment satisfaction, quality of life and general health status were included in the search protocol as it has been shown that all these areas might influence treatment adherence [18, 30]. The first search strategy included occurrences for QoL and treatment satisfaction including existing questionnaires known to the authors [((((((((((((Quality of life OR QoL OR Quality of life measurements OR Quality of life assessment) OR Quality of Life [Mesh]) OR euroqol eq-5d) OR sf-36) OR treatment satisfaction questionnaire) OR FLQA) OR DLQI) OR Skindex) OR PDI) OR psoriasis disability index) OR patient benefit index) AND ((validation) OR validity) OR reliability) AND psoriasis)], which provided 83 hits. Furthermore, the second search strategy included occurrences for adherence/non-adherence in combination with dermatology and topical treatment [((((predictor*) OR factor*) AND ((((complian*) OR non-complian*) OR adheren*) OR non-adheren*) AND ((((topic* therap*) OR topic* treatment*) OR dermatol* treatment*) OR dermatol* therap*)], which provided 40 hits. Citation titles, index terms, and abstracts from both searches were screened to identify potentially relevant articles containing or describing already existing tools, which were subsequently retrieved for full-text review [1–3, 5–9, 11, 12, 17, 19, 21–27, 31, 36, 39–42, 44, 46].

### Patient qualitative focus group interviews

In order to include all relevant aspects, needs and views of psoriasis patients, qualitative focus group interviews were conducted in February and March 2011, one with a national [4 (3 men and 1 woman) members of the German Patient’s Psoriasis Association—Deutscher Psoriasis Bund e.V] and an international [6 members (2 from Germany, 1 each from Spain, Denmark, Sweden, and The Netherlands) of the European Federation of Psoriasis Patient Organization—EUROPSO] patient advisory board. Patients participating in the interviews were selected according to their experience with topical treatment and in general with managing psoriasis and not according to their psoriasis condition. The aim was to have a representative population as the TTAQ questionnaire could be used for assessing adherence-influencing factors in all patients under topical treatments. Guided by an interviewer, participants were asked to give their feedback on the following topics: “important characteristics to be satisfied with topical treatment”, e.g., efficacy of treatment, side effects, “criteria to judge a topical treatment as practicable”, e.g., galenic and cosmetic properties, time expenditure required for application, how often should the medication be applied, “important factors for the appraisal of the value of a topical treatment”, e.g., expectations regarding the effect of the treatment, immediacy of beneficial effects and “important factors for being adherent with a prescribed topical treatment”, e.g., information received regarding the treatment and its correct application, recollection of this information, frequency of visits to the physician.

### Expert qualitative focus groups

A total of 11 experts from the fields of dermatology (7), psychology (1), health economic (1) and clinical research (2) were invited to participate in an expert panel meeting in April 2011. The corresponding experts reviewed each item of the developed item pool, identified via the literature search and confirmed during the patient interviews, and its relevance and suitability was evaluated for inclusion or exclusion from the newly developed TTAQ.

### TTAQ feasibility check

The 71-item containing TTAQ was initially assessed in a feasibility check with *n* = 59 psoriasis patients who were selected from different sites and who used topical treatments for psoriasis. The draft version of the questionnaire was sent to the patients along with a cover letter explaining them the scope for the development of this questionnaire and that their feedback is requested in order to perform an initial feasibility check and importantly to assess the comprehensibility of the items. Patients were asked to answer the TTAQ and to comment on the comprehensibility and relevance of the TTAQ items on a separate sheet. Patients were requested to fill-out the questionnaire anonymously and send it back to the authors per post. Item characteristics were analyzed descriptively by computing mean, standard deviation, range, skewness and kurtosis. Item difficulty and item–total correlation were also calculated for all items. Reliability of the TTAQ scales was assessed by computing internal consistencies (Cronbachs’s *α*) over all items.

## Results

### Literature search

Sixteen assessment tools were identified which were generic, dermatology specific or psoriasis specific. Out of these tools, 11 were considered as relevant to our research aims: the EuroQOL 5D [8], the Freiburg Life Quality Assessment (FLQA) [46], the Short Form 36 (SF36) and Short Form 12 (SF12) [11], the Dermatology Life Quality Index (DLQI) [23], the Skindex [7], the Treatment Satisfaction Questionnaire for Medication (TSQM) [2], the Psoriasis Disability Index (PDI) [11], the Patient Benefit Index (PBI) [6], the Belief and Behavior Questionnaire (BBQ) [16], the Brief Medication Questionnaire [33] and the Medication Adherence Self-Report Inventory (MASRI) [34]. These tools assessed either adherence-influencing factors or treatment satisfaction in patients under medication, or disease related or general quality of life in dermatological diseases or in psoriasis. In summary, these tools addressed the following areas: psyche, pain, symptoms, side effects, everyday coping, mobility, self assessment/health status treatment evaluation, overall satisfaction/condition, professional- and social life, choice of clothing, sports/leisure, love-life, satisfaction efficacy medication, satisfaction symptom relief, satisfaction time required, special questions regarding side effects, overall satisfaction medication, valuation/trust in medication, risk–benefit assessment, benefit assessment of treatment, cost of treatment and effect of treatment. After a careful examination, consideration and discussion of these constructs, the following domains were decided by the authors to be included in the newly developed TTAQ and to be placed under discussion by the qualitative patient and expert focus interviews: “Patient’s benefit from treatment”, “Knowledge, communication and relationship with the physician” and “Patient preference and satisfaction with the treatment”. The items within the domains “Patient’s benefit from treatment” and “Patient preference and satisfaction with treatment” were created with a special focus on topical treatment while the ones within the domain “Knowledge, communication and relationship toward the doctor” assess the quality of the patient–physician relationship as well as the amount of information the patients receive concerning their condition and its treatment with the relationship to the physician. Subsequently, the draft version of the TTAQ was created by formulating items within these domains that aimed at reflecting and assessing topical treatment adherence-influencing factors.

### Patient qualitative focus group interviews

The later analysis of all items collected and discussed during these interviews revealed that all aspects reported by the patients were already mentioned in the relevant literature and included in the item pool that had been created from the literature search.

### Expert qualitative focus groups

No relevant changes to the presented items were deemed necessary.

### TTAQ construction

A total of 71 items were decided to be included into the first version of the TTAQ. The TTAQ included now the four domains “Patient’s benefit from treatment”, “Knowledge, communication and relationship with the physician”, “Patient preference” and “Patient satisfaction with treatment”. All items were scaled in a four-point Likert format (0 = strongly disagree, 1 = disagree, 2 = agree and 3 = strongly agree), with a supplementary option to tick “Does not apply to me”.

### TTAQ feasibility check

Out of 89 patients with psoriasis to whom the TTAQ questionnaire was sent, 59 (66.3 %) completed and returned the questionnaire. Sociodemographic and medical baseline data of the patients were not considered.

Out of the 71 four-point ordinal-scaled items, 6 items showed a range lower than 3.00. Difficulties, item–total correlations and selection indices for each item are shown in Suppl. Table 1. Item difficulty should range between *D* = 0.20 and 0.80 [33]. Two items showed high difficulties (*D* < 0.20), i.e., it was very difficult to reach high values in these items. 14 items showed very low difficulties (*D* > 0.80), i.e., it was very easy to approach high ratings on these items.

The item–total correlation is defined as the correlation of responses to individual items with overall test score without the respective item. The higher the correlation, the more the item results are consistent with the scale as a whole. An insufficient item–total correlation is assumed if the item–total correlation *r*_itt_ is lower than 0.20 [33]. No item showed an item–total correlation of *r*_itt_ < 0.20. The lowest values showed the items 13 (*r*_itt_ = 0.20) and item 14 (*r*_itt_ = 0.26).

The Mittenecker and Ebel selection criterion (Sj) considers both the item–total correlation and the item difficulty and hence is regarded as a better evaluation criterion than the use of the item difficulty and item–total correlation alone. Items with Sj < 0.50 are regarded as less suitable [33]. As seen in Suppl. Table 1, two items (which also showed the lowest item–total correlation) did not reach this criteria (13, Sj = 0.24 and 14, Sj = 0.29).

Internal consistency was measured by Cronbach’s *α* over all items. All items had Cronbach’s *α* values that were higher than 0.80 and hence were considered as acceptable [25].

### Adjustments and modifications

From the item analysis, items 13 and 14 were found to be the least suitable; both showed the lowest item–total correlations and did not reach the Mittenecker and Ebel selection criteria. Therefore, both items were omitted from the final version of the questionnaire. In Table 1 the TTAQ and the PPQ domains and the ranges of the difficulty, item–total correlation and selection index are depicted.

**Table 1:** Difficulties, item–total correlations and selection indices of TTAQ and PPQ

Questionnaire/domain	Number of items	Difficulty	Item–total correlation	Selection index
TTAQ				
Patient benefit	40	0.12–0.85	0.42–0.92	0.52–1.10
Knowledge, communication and relationship with physician	7	0.78–0.86	0.58–0.92	0.82–1.26
Satisfaction with treatment	12	0.65–0.85	0.51–0.93	0.64–1.25
PPQ				
Patient preferences	10	0.63–0.77	0.60–0.97	0.65–1.14

On the basis of the patients’ evaluation on item comprehensibility and suitability, various expressions and wordings were adapted in order to reduce misunderstandings of the items. In addition, items 44–53 referring to the patient preference domain were decided to form a separate questionnaire (Patient Preference Questionnaire, PPQ, see Appendix 2), because a single assessment of the patient’s treatment preference between current and previous treatment seemed to be sufficient, while all the other domains included in the TTAQ should be assessed more than once during the patient’s current treatment with a topical medication.

The final version of the TTAQ contained 59 items that were divided into three domains (see Appendix 1). Table 1 shows for each of the four domains the number of all items that were included in the final version of the TTAQ and the PPQ and their range difficulties, item–total correlations and selection indices.

## Discussion

Favorable treatment outcomes are strongly dependent on medication adherence rates [16]. Importantly, it has been reported that adherence for patients in daily clinical practice is significantly different (i.e., lower) than the one observed in a clinical trial setting and hence not all patients benefit from a treatment as might be expected from the results of such clinical trials [18].

Therefore, it is of great importance to have reliable and easy-to-use tools which can be used in clinical practice for assessing predictors of treatment adherence and non-adherence. To date no such a tool exists which is routinely used in clinical practice for assessing topical treatment adherence-influencing factors in patients with dermatologic conditions. Given that dermatological conditions significantly impair the patient’s QoL and treatment regimens are often considered as time consuming, complicated and unpleasant, developing a novel tool that could assess these specific conditions and patient-related factors seems to be highly relevant. Additionally, the new tool enables to assess the relationship and information flow between the patient and the physician since this is considered to influence the patient’s treatment adherence to a major extent.

Those pillars supported the development of the TTAQ. The first preliminary results of the feasibility check indicate that the TTAQ contains psychometrically sound items which may be reliable for assessing factors of topical treatment adherence.

Importantly, both the TTAQ as well as the PPQ were translated from their German template in a validated way (forth and back translation) in seven languages: Danish, Dutch, English, French, Italian, Spanish and Swedish. Both questionnaires are currently being used in a multicenter randomized, controlled trial with appr. 2,000 psoriasis patients under topical treatment performed in Denmark, France, Germany, Italy, The Netherlands, Spain, Sweden and the UK. Within this pilot exploratory study, both questionnaires will be given to the patients at different time points during their treatment in order to assess when the questionnaires should be used, i.e., prior to starting a treatment or after a definite amount of time. In any case, the final versions of the tools would aim to identify adherence risk factors early on which would then enable the physicians to “predict” non-adherent behaviors from patients as well as the reasons which might lead to such behaviors. For example, if a patient would reply in questions 12–17 of the TTAQ that the current topical treatment limits his/her activities, then this might serve as a hint for the physician to discuss and address with the patient these issues and potentially even consider different treatment possibilities. Furthermore, following the evaluation of the results from the currently ongoing pilot study the number of items, especially in the TTAQ, will be re-considered. Since it is aimed that the newly developed tools will be used—once validated—in daily clinical practice, any redundant items will be deleted in order to reduce the time needed to fill-out the questionnaire and hence to increase its practicability. Summarizing the ongoing exploratory study mainly aims to further develop and fine-tune both tools. In accordance with the COSMIN taxonomy [37] the following validation criteria will be evaluated either during the currently ongoing exploratory pilot study or at a later time point: internal consistency, reliability, content validity, construct validity (including convergent and discriminant validity, hypotheses testing and cross-cultural validity), criterion validity, and responsiveness.

The aim of the future use of TTAQ in clinical practice is to allow physicians to identify potential factors for non-adherence at an early time point and to enable them in that way to apply adherence-enhancing interventions according to patient’s individual needs.

## Electronic supplementary material

Below is the link to the electronic supplementary material.

Supplementary material 1 (PDF 77 kb)

## Figures and Tables

**Table:**
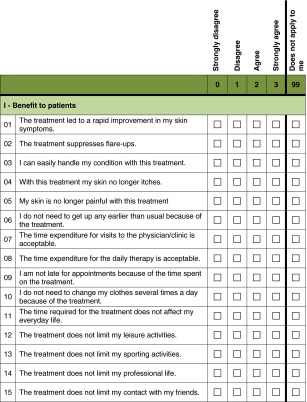

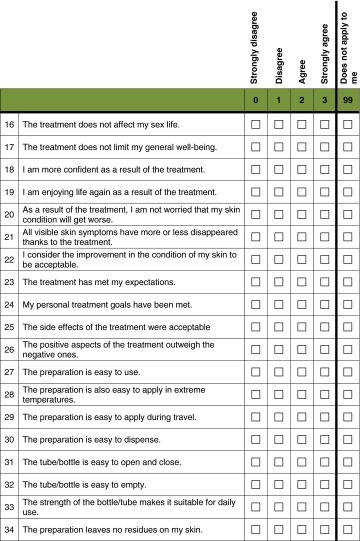

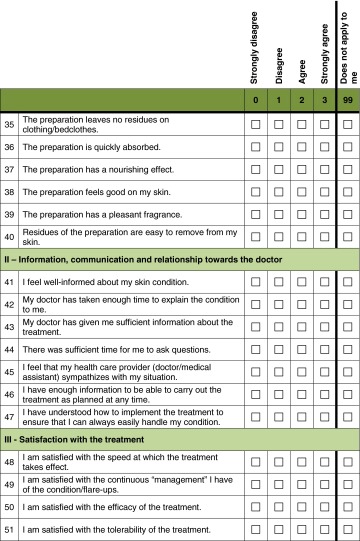

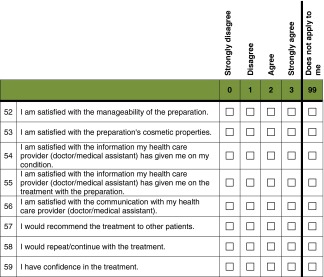
Final English Version of TTAQ

**Table:**
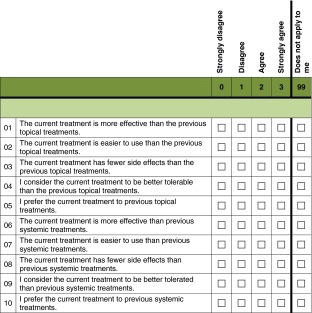
Final English Version of PPQ
