# Supplementary material for: Assessing adherence factors in patients under topical treatment: development of the Topical Therapy Adherence Questionnaire (TTAQ)
Source: Arch Dermatol Res. 2014 Feb 8;306(3):287–97. doi: 10.1007/s00403-014-1446-x (PMC3955139; doi:10.1007/s00403-014-1446-x)
Supplement: Supplementary file 1 — Supplementary material 1 (PDF 77 kb) [file 403_2014_1446_MOESM1_ESM.pdf]

Assessing Adherence Factors in Patients under Topical Treatment: Development of the Topical Therapy Adherence Questionnaire (TTAQ)

Archives of Dermatological Research

Ina Zschocke[a], Ulrich Mrowietz[b], Annett Lotzin[a], Eleni Karakasili[a], Kristian Reich[c*]

[a]SCIderm GmbH, Drehbahn 1-3, 20354, Hamburg, Germany

[b]Psoriasis-Center, Department of Dermatology, University Medical Center Schleswig-Holstein, Schittenhelmstraße. 7, 24105, Kiel, Germany

[c]Dermatologikum Hamburg, Stephansplatz 5, 20354, Hamburg, Germany

*Corresponding Author:
Kristian Reich
Dermatologikum Hamburg
Stephansplatz 5
20354 Hamburg
Germany
Telephone: +49-040-35 10 75 0
Fax: +49-40-35 10 75 10
Email: kreich@dermatologikum.de

Supplemental Table 1 Difficulties, Item-total Correlations and Selection Indices of the items of the TTAQ pilot version. Following the pilot test phase various expressions and wordings were adapted in order to reduce misunderstandings of the items. Each item's number at the final versions of TTAQ and PPQ is stated in the parentheses

Patient Benefit	n	Difficulty	Item-total Correlation	Selection index
ttq.ben_2 (ttaq_1)	53	0,61	0,86	0,88
ttq.ben_3 (ttaq_2)	53	0,63	0,90	0,93
ttq.ben_4 (ttaq_3)	55	0,66	0,79	0,83
ttq.ben_5 (ttaq_4)	52	0,63	0,79	0,82
ttq.ben_6 (ttaq_5)	46	0,70	0,79	0,86
ttq.ben_7 (ttaq_6)	49	0,20	0,85	1,05
ttq.ben_8 (ttaq_7)	54	0,80	0,55	0,69
ttq.ben_9 (ttaq_8)	52	0,79	0,42	0,52
ttq.ben_10 (ttaq_9)	53	0,12	0,86	1,33
ttq.ben_11 (ttaq_10)	55	0,20	0,70	0,88
ttq.ben_12 (ttaq_11)	52	0,22	0,76	0,91
ttq.ben_13	57	0,78	0,20	0,24
ttq.ben_14	57	0,71	0,26	0,29
ttq.ben_15 (ttaq_12)	57	0,80	0,50	0,63
ttq.ben_16 (ttaq_13)	54	0,85	0,62	0,87
ttq.ben_17 (ttaq_17)	49	0,85	0,55	0,77
ttq.ben_18 (ttaq_15)	57	0,85	0,75	1,06
ttq.ben_19 (ttaq_16)	54	0,83	0,79	1,06
ttq.ben_20 (ttaq_17)	58	0,83	0,79	1,05
ttq.ben_21 (ttaq_18)	54	0,68	0,79	0,85
ttq.ben_22 (ttaq_19)	49	0,66	0,93	0,98
ttq.ben_23 (ttaq_20)	56	0,62	0,80	0,82
ttq.ben_24 (ttaq_21)	57	0,57	0,92	0,93
ttq.ben_25 (ttaq_22)	57	0,48	0,90	0,90
ttq.ben_26 (ttaq_23)	55	0,56	0,92	0,93
ttq.ben_27 (ttaq_24)	56	0,50	0,82	0,82
ttq.ben_28(ttaq_25)	13	0,74	0,46	0,53
ttq.ben_29 (ttaq_26)	56	0,71	0,81	0,90
ttq.ben_30 (ttaq_27)	49	0,84	0,80	1,10
ttq.ben_31 (ttaq_28)	33	0,78	0,70	0,84
ttq.ben_32 (ttaq_29)	38	0,76	0,65	0,76
ttq.ben_33 (ttaq_30)	42	0,83	0,66	0,89
ttq.ben_34 (ttaq_31)	33	0,79	0,81	0,99
ttq.ben_35 (ttaq_32)	32	0,71	0,57	0,63
ttq.ben_36 (ttaq_33)	33	0,75	0,62	0,71
ttq.ben_37 (ttaq_34)	36	0,64	0,74	0,77
ttq.ben_38 (ttaq_35)	34	0,59	0,76	0,77
ttq.ben_39 (ttaq_36)	35	0,63	0,73	0,76
ttq.ben_40 (ttaq_37)	34	0,61	0,74	0,76
ttq.ben_41 (ttaq_38)	34	0,64	0,65	0,68

ttq.ben_42 (ttaq_39)	32	0,49	0,67	0,67
ttq.ben_43 (ttaq_40)	31	0,62	0,54	0,56
**Patient Preferences**	**n**	**Difficulty**	**Item-total Correlation**	**Selection index**
ttq.präf_44 (ppq_1)	39	0,71	0,97	1,07
ttq.präf_45 (ppq_2)	40	0,69	0,71	0,77
ttq.präf_46 (ppq_3)	35	0,63	0,87	0,90
ttq.präf_47 (ppq_4)	39	0,72	0,95	1,06
ttq.präf_48 (ppq_5)	42	0,75	0,98	1,14
ttq.präf_49 (ppq_6)	31	0,63	0,90	0,93
ttq.präf_50 (ppq_7)	31	0,70	0,60	0,65
ttq.präf_51 (ppq_8)	27	0,77	0,77	0,91
ttq.präf_52 (ppq_9)	31	0,68	0,90	0,96
ttq.präf_53 (ppq_10)	31	0,61	0,89	0,91
**Knowledge, Communication and Relationship with Physician**	**n**	**Difficulty**	**Item-total Correlation**	**Selection index**
ttq.kom_54 (ttaq_41)	59	0,80	0,70	0,87
ttq.kom_55 (ttaq_42)	59	0,78	0,78	0,94
ttq.kom_56 (ttaq_43)	58	0,84	0,87	1,18
ttq.kom_57 (ttaq_44)	59	0,85	0,92	1,28
ttq.kom_58 (ttaq_45)	59	0,86	0,88	1,26
ttq.kom_59 (ttaq_46)	55	0,85	0,58	0,82
ttq.kom_60 (ttaq_47)	51	0,85	0,84	1,18
**Satisfaction with Treatment**	**n**	**Difficulty**	**Item-total Correlation**	**Selection index**
ttq.zufr_61 (ttaq_48)	56	0,65	0,79	0,83
ttq.zufr_62 (ttaq_49)	52	0,72	0,86	0,96
ttq.zufr_63 (ttaq_50)	56	0,67	0,93	0,99
ttq.zufr_64 (ttaq_51)	56	0,73	0,71	0,80
ttq.zufr_65 (ttaq_52)	47	0,80	0,68	0,85
ttq.zufr_66 (ttaq_53)	35	0,69	0,77	0,83
ttq.zufr_67 (ttaq_54)	57	0,80	0,51	0,64
ttq.zufr_68 (ttaq_55)	57	0,83	0,88	1,17
ttq.zufr_69 (ttaq_56)	55	0,85	0,88	1,25
ttq.zufr_70 (ttaq_57)	56	0,72	0,86	0,96
ttq.zufr_71 (ttaq_58)	57	0,75	0,84	0,97
ttq.zufr_72 (ttaq_59)	57	0,74	0,91	1,03
